# *Cucumeropsis mannii* seed oil protects against Bisphenol A‐induced testicular mitochondrial damages

**DOI:** 10.1002/fsn3.3260

**Published:** 2023-02-07

**Authors:** Patrick Maduabuchi Aja, Hilary Akobi Ogwoni, Peter Chinedu Agu, Ejike Ugbala Ekpono, Joshua Nonso Awoke, Oliver Ugochukwu Ukachi, Obasi Uche Orji, Boniface Anthony Ale, Chinonso Peter Nweke, Ikechukwu Okorie Igwenyi, Esther Ugo Alum, Darlington C. Chukwu, Christian E. Offor, Atamgba Agbor Asuk, Ejike Daniel Eze, Ojochenemi E. Yakubu, J. B. Akobi, Onyedika Gabriel Ani, Chinaza Godswill Awuchi

**Affiliations:** ^1^ Department of Biochemistry Ebonyi State University Abakaliki Nigeria; ^2^ Department of Biochemistry Kampala International University Bushenyi Uganda; ^3^ Department of Biochemistry Mbarara University of Science and Technology Mbarara Uganda; ^4^ Department of Biochemistry University of Nigeria Nsukka Nigeria; ^5^ Department of Medical Biochemistry Cross River University of Technology (CRUTECH) Calabar Nigeria; ^6^ Department of Physiology Kabale University Kabale Uganda; ^7^ Department of Biochemistry Federal University Wukari Wukari Nigeria; ^8^ School of Natural and Applied Sciences Kampala International University Kampala Uganda

**Keywords:** antioxidants, Bisphenol A, *Cucumeropsis mannii*, mitochondria, systemic toxicity

## Abstract

There has been increasing search for the ameliorative properties of seed oils against toxicants. bisphenol A acts as an estrogenic endocrine‐disrupting chemical capable of causing male infertility. This study aimed to explore *Cucumeropsis mannii* seed oil effects against mitochondrial damage in rats using bisphenol A. Forty‐eight rats were randomly assigned to six groups (*n* = 6) of eight rats each and fed the same food and water for 6 weeks. The group A rats were given 1 mL olive oil, while the ones in group B were given bisphenol A at 100 mL/kg body weight via oral route. Group C received *C. mannii* seed oil 7.5 mL/kg body weight *C. mannii* seed oil, while group D, group E, and group F were pre‐administered bisphenol A at 100 mL/kg body weight, followed by treatment with *C. mannii* seed oil at 7.5, 5, and 2.5 mL/kg body weight, respectively. Antioxidant enzymes, glutathione, reactive oxygen species, testicular volume, malondialdehyde, body weight, and testicular studies were done using standard methods. The results of the bisphenol A‐administered group showed a significant decrease in the antioxidant enzymes, glutathione, body weight, and testicular volume with elevation in the levels of reactive oxygen species, malondialdehyde, and testicular indices. BPA + CMSO‐treated group showed a significant increase in GPx activity compared with BPA‐exposed rats. CMSO treatment significantly increased catalase activity in comparison with that of rats exposed to BPA. Remarkably, *C. mannii* seed oil and bisphenol A co‐administration significantly reversed the abnormalities observed in the dysregulated biochemical biomarkers. Our findings suggest that *C. mannii* seed oil has considerable antioxidant potential which can be explored in therapeutic development against systemic toxicity induced by exposure to bisphenol A. *Cucumeropsis mannii* seed oil protects against bisphenol A‐induced testicular mitochondria damages.

## Graphical abstract

Co‐administration of *Cucumeropsis mannii* seed oil and bisphenol A significantly reversed abnormal trends observed in the dysregulated biochemical biomarkers. This finding shows that *C. mannii* seed oil has considerable antioxidant potential which can be explored in therapeutic development against systemic toxicity induced by exposure to bisphenol A.

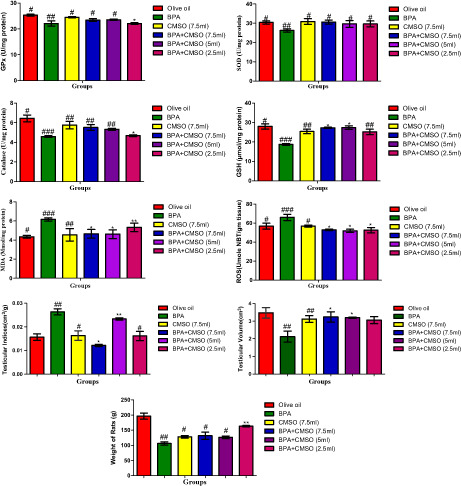

## 1 INTRODUCTION

Infertility has long been a global health barrier to successful reproduction for couples seeking to have one or more children. Infertility affects millions of people of reproductive age all over the world. Data estimate that between 48 million couples and 186 million people suffer infertility worldwide (WHO, 2021). Approximately 90% of male infertility is caused by reduction in sperm count and/or sperm quality (Gurunath et al., 2011). Male infertility accounts for 20%–30% of all infertility cases worldwide (Gurunath et al., 2011; Mohapatra et al., 2010). In sub‐Saharan Africa, male infertility is prevalent, ranging from 20% to 46%. Several factors contribute to sperm defects, including genetic abnormalities, environmental pollutants, smoking, hormone deficiency, and alcohol or cocaine use (Gurunath et al., 2011; Leslie et al., 2022). Recent data show that men are solely responsible for 20% of infertility cases worldwide and contribute to another 30%–40% of all cases of infertility (Leslie et al., 2022; WHO, 2021).

Bisphenol A (BPA) is among the most common environmental chemicals produced all around the world, accounting for approximately 6 billion pounds per year. Although it is used to produce epoxy resins and polycarbonate plastics, BPA is released from container walls by heat, thus penetrating the body system to exert genotoxic and cytotoxic effects (Awuchi & Awuchi, 2019; Dobrzyńska & Radzikowska, 2013; Geens et al., 2012). According to Sirasanagandla et al. (2022) and Mohapatra et al. (2010), BPA is an estrogenic endocrine‐disrupting chemical capable of causing male infertility. Furthermore, a review by Ranjit et al. (2010) reported that BPA affects sperm production, lowers serum testosterone levels, and thins the seminiferous epithelium. Takahashi and Oishi (2003) also discovered that BPA affects the activities of testicular mitochondria. The interaction of BPA with androgen and estrogen receptors causes reproductive toxicity (Cimmino et al., 2020; Sirasanagandla et al., 2022). After high subcutaneous and oral dosing, BPA may also potentiate uterotrophic effects (Cimmino et al., 2020; Sirasanagandla et al., 2022). Because of its endocrine‐disrupting properties, BPA can cause oxidative stress, resulting in the generation of reactive oxygen species such as superoxide radicals, hydroxyl radicals, and hydrogen peroxide (Awuchi & Awuchi, 2019; Gurunath et al., 2011).

*Cucumeropsis mannii* is indigenous to tropical Africa, especially in the West of the Great Rift Valley. It is cultivated for food and as a source of oil in this tropical region (Agu et al., 2022). In addition to being known as white‐seed melon and Mann's cucumeropsis in English, it is also known as ahu‐ilu in Igbo, agushi in Hausa, and elegushi in Yoruba (Koffi et al., 2008) in Nigeria. It has climbing vines that may reach a length of 4 m and are coated with stiffened hair‐like structures. The roughly palmate or heart‐shaped leaves may reach lengths of 12 cm and widths of 14 cm (Mbuli et al., 1983). It has tiny yellow petals that are around a centimeter long on both the male and female flowers. About 19 cm long and 8 cm broad, the fruit is egg‐shaped or elongated oval in form. The fruit has a creamy texture and traces of green. The fruits and seeds can both be consumed and also employed for various industrial processes and products (Okwundu et al., 2021). In west African countries, various parts of the *C. mannii* plants have been utilized (Agu et al., 2022; Okwundu et al., 2021). Ghanaians use the juice from the flesh fruit concocted with various condiments and apply to navel of newly born infants to fast‐track the curing progression till cord vestiges drip off; equally, in Gabon, steeped leaves have been in use as a purgative to constipated suckling babies (Agu et al., 2022). The Sierra Leonian cattle boys conventionally make use of the desiccated *C. manni* fruit shell as a horn warner (Awuchi, 2023; Leakey et al., 2022). The kernel of the seed contains semi‐drying oils for making soap, cooking, lighting, livestock feed, and the fruit for preparing healing ointment (Koffi et al., 2008; Okwundu et al., 2021; Olarewaju et al., 2021).

Similarly, research has revealed that *C. mannii* seeds are a good source of essential amino acids, essential fatty acids, minerals, vitamins, polyphenols, and other important phytochemicals, which are nutraceuticals explorable in tackling various illnesses (Basharat et al., 2023; Kortse & Oladiran, 2013; Okwundu et al., 2021). For instance, the essential fatty acids such as oleic, linoleic, stearic acid, and omega‐6 fatty acids which are the major components of the *C. mannii* seeds that have antimicrobial, antioxidant, and anti‐inflammatory properties, explaining their therapeutic potentials against infections such as gastrointestinal tract infections, infertility, oxidative damage, etc. (Kortse & Oladiran, 2013; Nwozo et al., 2023). The characteristic phytochemical constituents reported present in *C. mannii* seed and its oil according to Badifu and Ogunsua (1990) were alkaloids, phenols, flavonoids, terpenes, and tannin, which are exogenous antioxidants. Agu et al. (2022) reported that *Cucumeropsis mannii* seed oil (CMSO) reversed altered testicular histology/biochemistry in male rats against toxicity induced by BPA. We considered that there is no limit to the exploration of plants' therapeutic potentials (Aja et al., 2014) and adopted the use of animal model to further enlist *C. mannii* seed oil potentials as an emerging therapeutic natural product. Therefore, the present study investigated the effects of *C. mannii* seed oil (CMSO) against mitochondrial damage induced by bisphenol A in male albino rats. The study demonstrated that CMSO is suitable as a nutritional and pharmacological alternative against infertility caused by exposure to toxicants such as BPA.

## 2 MATERIALS AND METHODS

### 2.1 Chemicals

Bisphenol A was obtained from Sigma‐Aldrich Corp (Trusca et al., 2019). trichloroacetic acid (TCA), Bovine serum albumin (BSA), 3,4,3‐(4,5‐dimethylthiazol‐2‐yl)‐2,5‐diphenyltetrazoliumbromide (MTT), 2,7‐dichlorofluorescein diacetate (DCFH‐DA), thiobarbituric acid (TBA), reduced glutathione, 1,1,3,3‐tetramethoxypropane, Coomassie Brilliant Blue powder, and oxidized glutathione were obtained from Sigma‐Aldrich. Sucrose 5, 5′‐dithiobis‐2‐nitrobenzoic acid (DTNB), NaHCO_3_, NaCl, MgCl_2_, KCl, CaCl_2_, and dimethyl sulfoxide (DMSO) were purchased from Merck Company. Assay kit of glutathione (GSH; Cat. No. S0052) and glutathione peroxidase (GPx; Cat No. S0058) were obtained from Beyotime Institute of Biotechnology.

### 2.2 Collections and authentication of plant materials

The *C. mannii* (Abakaliki wild type: Egusi) seed was obtained from Iboko market, Izzi Local Government Area, Ebonyi state, Nigeria. The plant was authenticated and classified by Mr. O. E. Nwankwo, a plant taxonomist at the Applied Biology Department, Ebonyi State University, Ebonyi state, Nigeria. The method used by Agu et al. (2022) were followed.

### 2.3 CMSO extraction

The seeds of *C. mannii* were peeled and ground using automated grinder. Seed oil was then locally extracted using mechanical pressing with the help of mortar and pestle (Ferrentino et al., 2020). Water drops were introduced to improve oil release as the water helps in the rupturing of the cells, partly by binding to gum/mucilage (hydrocolloids; Dror et al., 2006), which are allowed to sediment. The extracts were stood for 2–3 days undisturbed to sediment and was then separated by decantation to recover oil with purer quality. The oil was then kept in clean bottles for use.

### 2.4 Acute toxicity of CMSO

CMSO acute toxicity in Wistar Albino rats (male) was evaluated according to OECD (2008) as specified in the guideline No. 425. Acute toxicity was done using the test procedure of limit dose according to the guideline No. 425. For this experiment, 2 months old male albino Wistar rats were employed for the animal study and were acclimatized in the laboratory for 7 days before the experiment. Fifty mL/kg of CSMO was orally given to a female rat after fasting overnight. Thereafter, the rat was strictly monitored for any observed behavioral or physical change for initial 30 min after extract's administration, and then periodically observed for the next 24 h, with more attention within the first 4 h, and then monitored on daily basis for 14 days. Foods were given to the rats after 3–4 h administration of CMSO. After the first rat survival, the other four rats (male) were recruited, then fasted for 4 h. The rats subsequently received the same CMSO dose followed by same observation/monitoring, which continued for additional 14 days to watch for possible toxicity (Eleazu et al., 2021; Saleem, 2017; Tadesse et al., 2014). At 50 mL/kg limit test dose, there was no signs of gross behavioral or physical changes in the rats, such as motor activity, reduction in feeding, or hair erection during the 24 h and 14‐day monitoring periods. Consequently, 5 mL/kg limit dose (10%) was chosen as intermediate/middle dose, 2.5 mL/kg (half of this) was chosen as lower dose, while 7.5 mL/kg (1.5 times the middle dose) was chosen as the higher dose according to the guideline of OECD (2008) as stated in the OECD guideline No. 425.

### 2.5 Experimental rats

The animals used for the study were adult albino Wistar rats obtained from the Animal House, University of Nigeria Nsukka, Enugu, Nigeria, and weighed 190–350 g. Rats not within this experimental range were not used. The experimental rats were obtained and kept in rat cages made of stainless steel in ventilated animal house at the Department of Biochemistry, Ebonyi State University, Abakaliki, Nigeria. The rats were allowed 7 days acclimatization under good sanitary/laboratory conditions at room temperature and 12 h dark/light cycle (Arias‐Reyes et al., 2021; Ewere et al., 2021). The rats were allowed unrestricted access to Vital Feed® and water. The feed was a product of Grand Cereals Limited, Jos, Nigeria. The International Standard Procedure for Experimental Animal Handling of the National Institute of Health (NIH), USA (NIH Publications No. 80–23, revised in 1996), which has been adopted by the Department of Biochemistry, Ebonyi State University, Abakaliki, Nigeria, was followed with approval by the Ethical Committee in the Department, with ethical approval number EBSU/BCH/ET/19/001.

### 2.6 Experimental design

Total of 48 male albino Wistar rats were recruited for the experiment and randomly grouped into six groups labeled group A, group B, group C, group D, group E, and group F, with each group having eight rats. Group A, group B, and C were used as control, while group D, group E, and F received the treatment (Table 1).

**TABLE 1:** Rat groups.

Group	Administration
A	Only 1 mL olive oil. Served as normal control as control group 1 (CG1)
B	Oral 100 mg/kg BW BPA. Control for BPA; CG2 (control group 2)
C	Oral CMSO 7.5 mL/kg BW. CMSO control. CG3 (control group 3)
D	BPA pre‐administered at 100 mg/kg BW and treated with CMSO at 7.5 mL/kg BW. Treatment group 1 (TG1)
E	BPA pre‐administered at 100 mg/kg BW and treated with CMSO at 5 mL/kg BW. Treatment group 2 (TG2)
F	BPA pre‐administered at 100 mg/kg BW and treated with CMSO at 2.5 mL/kg BW. Treatment group 3 (TG3)

CMSO and BPA administration was simultaneously given once per day by oral intubation for 6 weeks.

### 2.7 Collection of tissue sample

The sacrificing of the rats was done under mild anesthesia by cervical dislocation. Scrotal sacs were dissected using a sharp blade to remove the right and left testicles (Garcia & Sajjad, 2022). Their volume, length, width morphology was studied for all the groups. The calculation of testicular volume and indices was done using:

$$\text{Volume}=\left(D2/4\times \pi \right)\;L\times K.$$

Where, *L* = length, *D* = width, and *K* = 0.9.

### 2.8 Testicular mitochondria isolation

The testes were minced in freshly isolated medium (70 mM sucrose, 220 mM mannitol), 10 mM 2‐[4‐(2 hydroxyethyl) piperazin‐1‐yl] ethane sulfonic acid (HEPES), 1 mM Ethylenediaminetetraacetic acid (EDTA) buffer at pH 7.4, 2.5 mM MgCl_2_ (SRL), 0.5 mM. The centrifugation of the homogenate was done for 10 min at 500 *g*. The supernatant was retained, while a fresh isolation medium was used to wash the pellet and then recover using the initial supernatant (Bello et al., 2021; Park & Pang, 2021). The centrifugation of the pooled fractions was done for 10 min at 500 *g* and supplemented with protease and phosphatase inhibitors. The obtained supernatant was centrifuged for 15 min at 5000 *g* with the aim obtaining the mitochondria pellet. The pure mitochondria were recovered using centrifugation at 4°C for 10 min at 12,000 *g*. The supernatant containing mitochondria was collected and used for various biochemical analyses. (Bello et al., 2021; Park & Pang, 2021; Sayeed et al., 2006).

### 2.9 Antioxidant Enzymes' measurement

*GPx* was determined using Flohe and Gunzler (1984) method. A suspension of fresh isolation of mitochondria was made in 1 mL of buffer (50 mM Tris–HCl, 0.22 mM NADPH (Sigma), 1 mM GSH (Sigma), 5 mM EDTA, and 0.4 U glutathione reductase (Sigma) (pH 7.6)). The initiation of the reaction was done by the addition of Spectrochem (tertiary butyl hydroperoxide) to 0.22 mM final concentration, and absorbance was read at 340 nm. The enzyme activity was calculated as nmol NADPH oxidized/min/mg protein using a molar extinction coefficient of 6.22 × 10^3^ M^−1^ cm^−1^.

*The catalase activity assay* was performed according to the method reported by Khodayar et al. (2018). In brief, suspensions of mitochondria were added to H_2_O_2_ (0.01 M) and Tris–HCl (0.05 mM), mixed, and then incubated for 10 min. After which 4% ammonium molybdate was added. Absorbance was determined at 410 nm.

*Superoxide dismutase* was determined using Flohe and Otting (1984) method. Sonication was used to disrupt the 1 mg mitochondrial pellet suspension in 0.1 mM EDTA and 50 mM phosphate buffer (pH 7.8). After centrifugation, the supernatant was collected and analyzed for MnSOD activities. The mixture of the assay contains a sample, reaction buffer (0.5 M xanthine (Sigma), 0.1 mM NaOH (E. Merck), and 2 lM cytochrome c (SRL) in 0.1 mM EDTA and 50 mM phosphate buffer, at pH 7.8). The initiation of the reaction was done with the addition of 0.2 U/mL in 0.1 mM EDTA (xanthine oxidase (Sigma)). The absorbance change was read at 438 nm for 3 min. Activities of SOD were determined as its inhibitory ability against 50% ferricytochrome c decrease and presented as U/min/mg protein.

*The GSH level* was determined according to the Umapathy et al. (2018) description. Hence, testicular homogenization (1 mL) was incubated with EDTA (1 mL) and 20% TCA (1 mL) for 5 min. It was then centrifuged for 30 min at 4°C at 1000 rpm. Next, a mixture of the supernatant (200 μL) and 1.8 mL DTNB was made. GSH reaction with DTNB leads to the formation of yellow solution. Absorbance reading was measured at 412 nm. The values of the GSH are calculated as nmol/mg mitochondria protein.

*LPO* was determined according to the Buege and Aust (1978) description. The preparation of mitochondria (4 mg/mL) was heated in boiling water for 15 min with an equal Buege and Aust reagent (thiobarbituric acid (TBA) 0.37% w/v in 0.25 M HCl and TCA 15% w/v in 0.25 M HCl). After allowing to cool, the removal of the precipitate was done using centrifugation at room temperature for 10 min at 1000 *g*. Absorbance reading was taken at 532 nm.

Quantification of TBARS was done using 1.56 × 10^5^ M^−1^ cm^−1^ extinction coefficient and expressed in nmol of TBARS/mg of mitochondrial protein.

*ROS* was measured using the methods of Jain et al. (2011) and Ahangarpour et *al*. (2018). Exactly 100 L of tissue homogenates was incubated for 15 min at 37°C with the assay media (20 mM Tris–HCl, 13 mM KCl, 5 mM MgCl_2_, 20 mM NaH_2_PO_4_, 3.0 mM glucose, and 5 M DCF‐DA). The DCF formation was calculated at 488 nm excitation wavelength and 510 nm emission wavelength for 10 min using HITACHI fluorescence spectrometer (Model No. F7000) furnished with a FITC filter.

### 2.10 Statistical analysis

The statistics was done using GraphPad Prism 5.04 (GraphPad). The data were presented as mean ± standard deviation. ANOVA (one‐way) with Tukey's test was done. Generally, the significance level was *p* < .05.

## 3 RESULTS

**FIGURE 1:**
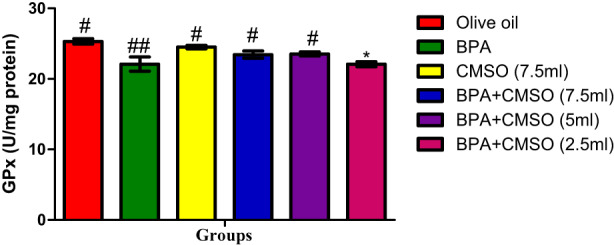
CMSO effects on glutathione peroxidase activity of testicular mitochondria in testicular toxicity induced by BPA in the albino rats. Results are calculated as mean ± standard deviation (*n* = 6). Mean with different signs have significant difference (*p* < .05).

As shown in Figure 1, BPA induction caused a significant decrease in glutathione peroxidase activities in comparison with control rats. The group of rats treated with CMSO (BPA + CMSO) showed a significant increase in GPx activity when compared to that of BPA‐exposed animals.

**FIGURE 2:**
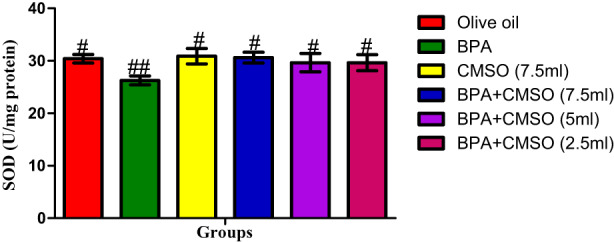
CMSO effects on superoxide dismutase activity in testicular mitochondria in testicular toxicity induced by BPA in the albino rats. Results are calculated as mean ± standard deviation (*n* = 6). Mean with different signs have significant difference (*p* < .05).

Figure 2 showed that SOD activity was significantly decreased on induction with BPA when compared to the normal control group. Interestingly, CMSO treatment showed a significant increase in SOD activity when compared to that of BPA‐exposed rats.

**FIGURE 3:**
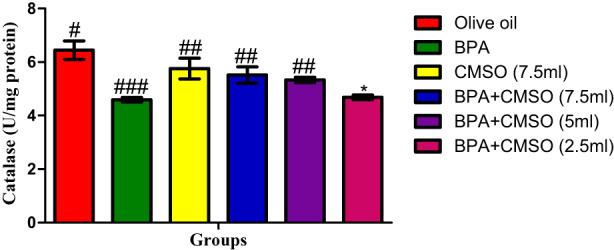
CMSO effects on catalase activity in testicular mitochondria in testicular toxicity induced by BPA in the albino rats. Results are calculated as mean ± standard deviation (*n* = 6). Mean with different signs have significant difference (*p* < .05).

Catalase activity as shown in Figure 3 significantly decreased in group exposed to BPA in comparison with the normal control. Conversely, CMSO treatment significantly increased catalase activity in comparison with that of rats exposed to BPA.

**FIGURE 4:**
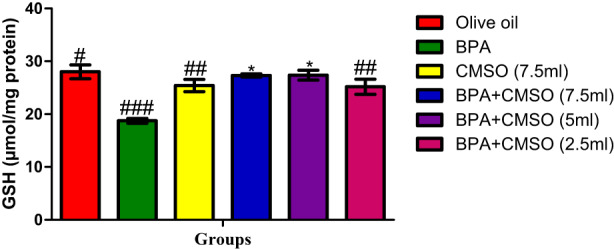
CMSO effects on reduced glutathione level in testicular mitochondria in testicular toxicity induced by BPA in the albino rats. Results are calculated as mean ± standard deviation (*n* = 6). Mean with different signs have significant difference (*p* < .05).

Figure 4 revealed that GSH level was significantly decreased in rats exposed to BPA in comparison with the normal control. However, CMSO treatment significantly increased and restored GSH levels when compared to BPA‐exposed rats.

**FIGURE 5:**
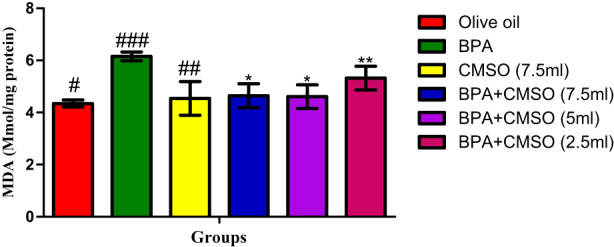
CMSO effects on malondialdehyde level in testicular mitochondria in testicular toxicity induced by BPA in the albino rats. Results are calculated as mean ± standard deviation (*n* = 6). Mean with different signs have significant difference (*p* < .05).

As shown in Figure 5, BPA administration significantly increased MDA level in the testicular mitochondria in comparison with the normal control. Interestingly, treatment with CMSO (BPA + CMSO) showed a significant decrease in the level of MDA when compared to the BPA group.

**FIGURE 6:**
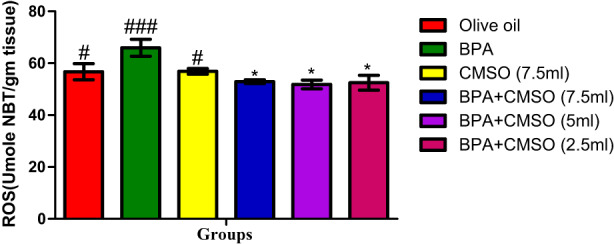
CMSO effects on reactive oxygen species level in testicular mitochondria in testicular toxicity induced by BPA in the albino rats. Results are calculated as mean ± standard deviation (*n* = 6). Mean with different signs have significant difference (*p* < .05).

Figure 6 revealed ROS level significantly increased in testicular mitochondria of BPA‐treated rats over the normal control. Rats in BPA + CMSO‐treated group had a significant reduction in ROS level when compared to the BPA‐treated group.

**FIGURE 7:**
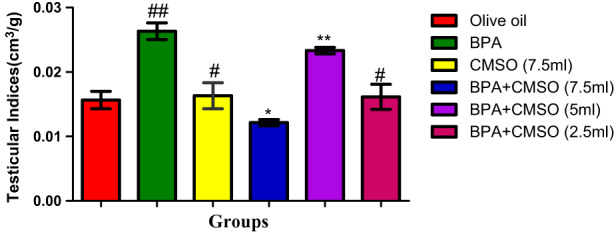
CMSO effects on testis indices in testicular homogenate in testicular toxicity induced by BPA in the albino rats. Results are calculated as mean ± standard deviation (*n* = 6). Mean with different signs have significant difference (*p* < .05).

As shown in Figure 7, a significant increase in testicular indices was observed in BPA‐treated rats over the normal control. BPA + CMSO‐treated rats presented a significant decrease in testicular indices when compared to the BPA‐treated group.

**FIGURE 8:**
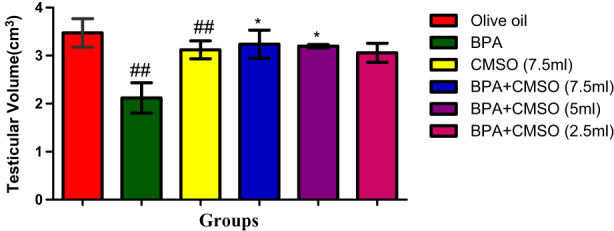
CMSO effects on testis volume of testicular homogenate in testicular toxicity induced by BPA in the albino rats. Results are calculated as mean ± standard deviation (*n* = 6). Mean with different signs have significant difference (*p* < 0.05).

Figure 8 revealed that BPA administration significantly decreased the level of testicular volume when compared to the normal control group. CMSO treated group (BPA + CMSO) had a significantly increased level of testicular volume in comparison with the BPA group.

**FIGURE 9:**
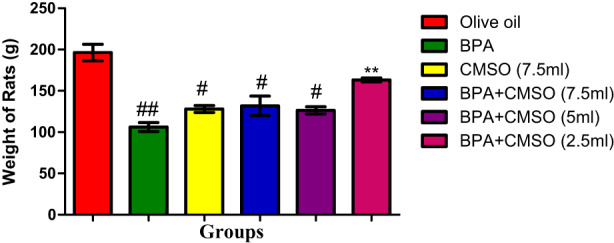
CMSO effects on bodyweight of testicular toxicity induced by BPA in the albino rats. Results are calculated as mean ± standard deviation (*n* = 6). Mean with different signs have significant difference (*p* < .05).

BPA treatment as shown in Figure 9 resulted in significantly decreased BW of rats in comparison with the normal control. CMSO group (BPA + CMSO) showed a significant increase in the rats' BW in comparison with that of the BPA‐treated group.

## 4 DISCUSSION

Bisphenol A, a well‐established endocrine disruptor, has been shown to induce cell permeability and enzyme inactivation (Catala, 2009). Sakaue et al. (2007) reported that BPA caused a decrease in antioxidant enzymes (GPx, SOD, and CAT), sperm count, and motility, and also affected the sperm morphology of adult male rats. Santiago et al. (2021) acknowledged the need to fight against BPA‐induced toxicity and recommended natural antioxidants as potential solution. The present study showed that BPA significantly decreased the activities of antioxidant enzymes such as GPx, SOD, and CAT. Similarly, the result is consistent with the work of Meeker et al. (2010), who reported that BPA caused a decline in antioxidant enzymes, semen quality, increased sperm DNA damage, and decreased spermatogenesis. Furthermore, the present results correlated with Bindhumol et al. (2003), who demonstrated that BPA drastically showed oxidative stress in the liver of male rats and drastically reduced liver enzyme activities.

Furthermore, these present results also agreed with the observation of Geens et al. (2012), who evaluated the efficiency of Naringin against testicular toxicity induced by BPA in adult rats and reported a significant decrease in GPx, SOD, and CAT. Therefore, the reduction in the activities of these enzymes (GPx, SOD, and CAT) may be due to the accumulation of reactive oxygen species (ROS) that may ultimately cause rapid oxidative damage to lipids, proteins, and DNA, thereby altering the physiological and biological functions of these enzymes. However, co‐administration of CMSO restored and significantly increased the activities of the antioxidant enzymes (GPx, SOD, and CAT). The increase in the activities of these enzymes may be due to the therapeutic efficacy and protective bioactive compounds present in seeds and seed oil (Awuchi & Okpala, 2022) such as those present in the CMSO. According to Kortse and Oladiran (2013), CMSO contains micronutrients such as vitamin E and vitamin A. The antioxidant defense system depends heavily on these vitamins. Antioxidants are found all around the cell and guard it against ROS harm. The antioxidant enzymes displayed decreased activity in the BPA‐treated animals. This decrease in enzyme activity suggests that BPA is having even more negative effects by aggravating the already compromised mitochondrial bioenergetics. SOD, CAT, and GPx lessen ROS oxidative damage to testicular cell membranes and shield the biological system from their negative effects. The CAT enzyme is inhibited when SOD activity is decreased because superoxide radicals may build up as a result of the change. Reducing CAT activity lowers the capacity of the testicles to remove hydrogen peroxide (H_2_O_2_) created following exposure to BPA. Lipids, proteins, and DNA can quickly suffer oxidative damage at the hands of H_2_O_2_ (Nwozo et al., 2023; Zahnit et al., 2022). Additionally, the GPx may directly function as an enzyme that inhibits sperm lipid peroxides and H_2_O_2_ by acting as an antioxidant. Rats treated with BPA showed a drop in GPx levels, which may indicate increased H_2_O_2_ generation and decreased GSH levels.

Furthermore, we reported that GSH levels significantly decreased in the testicular mitochondria of rats treated with BPA. This result correlates with the findings of Hassan et al. (2012), who reported that BPA caused a significant decline in the levels of GSH along with a decrease in the activity of SOD and CAT. Hence, the results of this present study further agreed with the results of Wu et al. (2011), who reported that low and high doses of oral administration of BPA revealed a significant decrease in the activities of GSH, CAT, and SOD in the liver and testis tissues of male rats. The reduction in GSH levels demonstrated the accumulation of free radicals and decreased activity in antioxidant enzymes and their application in free radical detoxification (Boubekeur et al., 2022; Nwozo et al., 2023). However, co‐administration of CMSO and BPA restored and significantly increased GSH levels to near normal, which may be due to the reducing properties and potential of CMSO.

More so, our study showed a remarkable increase in testicular lipid peroxidation in the BPA‐treated group. This result is consistent with the observation of Biedermann et al. (2010), who affirmed that BPA administration caused rapid degradation of lipids that ultimately led to the high level of MDA in the test sample, thus generating oxidative stress in testicular tissue. In furtherance to this, Li et al. (2012) reported that BPA declined the activity of the males' specific cytochrome P450 isoforms (testosterone, 2, and 6β hydroxylase), thus provoking ROS that impairs sperm function. Furthermore, the present study is consistent with the study of Kabuto et al. (2003), who reported that BPA administration significantly decreased the level of testosterone and progesterone and led to the accumulation of ROS.

The buildup of ROS that may have triggered the oxidative breakdown of lipids and resulted in the rise in MDA levels shown in the current study is one possible explanation. Free radicals may be able to steal electrons from the lipids in cell membranes as a result of these processes, damaging the cells. This process is carried out by a free radical chain reaction, which has the potential to further impact polyunsaturated fatty acids with many double bonds between methylene bridges (–CH_2_) that contain reactive hydrogen atoms.

Therefore, co‐administration of CMSO displayed anti‐lipid peroxidation activity and significantly decreased the level of MDA in testicular mitochondria. The therapeutic effectiveness and antioxidant qualities of CMSO, whose mechanism of action involves donating electrons, may be responsible for the restoration of the structural and functional integrity of membrane phospholipids. This mechanism can stop the electron chain reaction mechanism and neutralize the ROS generated by BPA. Since the mitochondrial membrane includes a sizable quantity of polyunsaturated fatty acids in its phospholipids, a rise in LPO signals that the mitochondria are producing reactive oxygen species. These polyunsaturated fatty acids and their byproducts have a variety of functions, including regulating gene expression, cell signaling, membrane structure, and energy production. Oxidative stress can cause lipid peroxidation, which can affect processes (Catala, 2009). Once more, Catala (2009) showed that BPA results in a drop in intracellular ATP, which promotes sperm motility. Generally speaking, spermatozoa are prone to damage because of the release of unsaturated fatty acids and their capacity to produce ROS.

Furthermore, BPA significantly increased the level of ROS in testicular mitochondria. Anjum et al. (2011) reported a similar deleterious increase in ROS level in bisphenol A‐induced biochemical toxicity in the mouse's testicular mitochondria. Our results also correlated with the study of Gassman (2017), who reported that BPA‐induced toxicity significantly increased the level of ROS, such as superoxide anion, hydroxyl radical (OH·), and singlet oxygen (^1^O_2_). These findings supported the results of Biedermann et al. (2010), who found that BPA administration caused rapid lipid peroxidation and an accumulation of free radicals, resulting in a high level of MDA in the test sample and oxidative stress in testicular tissue. This result is also consistent with the findings of Marieb and Hoehn (2014) that BPA caused toxicity and perturbation of the cell membrane that ultimately impaired MMP via the accumulation of ROS. The increase in ROS may be due to the partial reduction of oxygen. This increase in ROS could result in the formation of superoxide anions and the subsequent leakage of electrons from the various complexes in electron transport chains due to the disruption of mitochondrial membrane phospholipids (Novo & Parola, 2008).

However, co‐treatment of CMSO significantly decreased the level of ROS, which is most probably due to the therapeutic potential and reducing ability of CMSO or perhaps due to mitochondrial GSH's effects on the inner membrane permeability and sulphydryl group in the reduced state. Its metabolic implication is the impairment of the electron transport chain, thus inhibiting oxidative phosphorylation that may ultimately alter cellular metabolism and decrease sperm motility, thus resulting in infertility. This increase in ROS could result from the leakage of electrons at complex I and complex III from electron transport chains that lead to a partial reduction of oxygen and the formation of superoxide anions (Messaoudi et al., 2022; Novo & Parola, 2008). Collectively, both superoxide and hydrogen peroxide generated in this process is considered mitochondrial ROS (Turrens, 2003). During oxidative phosphorylation (OXPHOS), the electron transport chain inside the inner mitochondrial membrane generates most of the mitochondrial ROS (Awuchi & Twinomuhwezi, 2021; Nolfi‐Donegan et al., 2020).

The rats' body weight and testes were both drastically reduced by BPA, while testicular indices were noticeably elevated. This result is consistent with observations made by Gurmeet et al. (2014), who found that rats exposed to BPA saw a rise in the testicular index and a concurrent decrease in testicular volume and body weight. Similar findings have been reported for  BPA‐exposed rats that had significantly lower testicular volume and body weight than their control counterparts. Numerous studies have demonstrated that following the treatment of BPA, the body weight and testicular volume of male rats significantly decreased, with a concomitant rise in testicular indices (Korkmaz et al., 2010; Nanjappa et al., 2012; Norazit et al., 2012).

However, the significant decrease in testicular volume and BW with a corresponding increase in testicular indices observed in this study may be caused by reduced gender hormones' bioavailability, pointing to the males' reproductive endocrine stipulation. The volume and weight of testis have direct relationship with the spermatogenic cells' mass. Consequently, reduced volume and mass of testis affect the activity of spermatogenesis with a decrease in germ cells. Additionally, it is pertinent to note that intoxication with BPA reduced the BW of the experimental rats by exerting damages on key molecules, including proteins of testis. However, co‐treatment with CMSO significantly increased the BW and testicular volume with a concomitant decrease in testicular indices of male rats, which may be due to the therapeutic efficacy and protective potential of CMSO. As a result, our findings confirmed the previous observation by Anjum et al. (2011) that there was a significant decrease in body weight and testicular volume as well as an increase in testicular indices in BPA‐exposed groups. However, pre‐treatment of melatonin markedly increased testicular weight and volume, with a decrease in testicular indices.

Based on the findings from the present study, BPA exposure resulted in systematic impairments in the testicular mitochondria of male rats. It caused a dysregulation in the activities and levels of markers of oxidative damage investigated in this study. Prolonged exposure to BPA resulted in a drastic decrease in the body weight of rats, testicular volume, and a marked increase in testicular indices. Interestingly, the administration of *C. mannii* seed oil (CMSO) significantly normalized these toxic effects of BPA in all cases of markers investigated. Therefore, CMSO is an emerging nutraceutical of importance against systematic toxicity induced by exposure to BPA.

## 5 CONCLUSION

This study explored the effects of *C. mannii* seed oil against mitochondrial damage in albino rats using bisphenol A (BPA). The results of the BPA‐administered group show a significant decrease in the antioxidant enzymes, reduced glutathione, body weight, and testicular volume with elevation in the levels of reactive oxygen species, malondialdehyde, and testicular indices. The co‐administration of *C. mannii* seed oil and bisphenol A significantly reversed the abnormal trends observed in the dysregulated biochemical biomarkers, and ameliorated the toxicity induced by bisphenol A. *Cucumeropsis mannii* seed oil's redox imbalance modulation showed protective activities against bisphenol A‐induced testicular mitochondria damages. This plant and its oil can be subjected to clinical trials against infertility and other toxicities induced by bisphenol A and similar toxic compounds with similar chemical structures. Additional studies should be done to explore the specific components of the oil responsible for its specific biological activity. This may include exploring the areas not covered in this study, including screening bioactive components of CMSO against toxicities induced by BPA and other toxic substances.

## FUNDING INFORMATION

No funding was obtained for this study.

## CONFLICT OF INTEREST STATEMENT

The authors declare that there is no financial or non‐financial conflict of interest in the study.

## ETHICAL APPROVAL

In this study, we received ethical approval number EBSU/BCH/ET/19/001 from the Ethical Committee of the Biochemistry Department, Ebonyi State University, Nigeria, and followed their recommendations as adopted from the International Standard Procedure for Experimental Animal Handling of the National Institute of Health (NIH), USA (NIH Publications No. 8023, revised in 1996).

## INFORMED CONSENT

Not applicable.

## CONSENT FOR PUBLICATION

All the authors on this manuscript gave their consent to publish.

## Data Availability

Additional data will be made available on request.

## References

[fsn33260-bib-0001] Agu, P. C. , Aja, P. M. , Ekpono, U. E. , Ogwoni, H. A. , Ezeh, E. M. , Oscar‐Amobi, P. C. , Atamgba, A. A. , Ani, O. G. , Awoke, J. N. , Nwite, F. E. , Ukachi, O. U. , Orji, O. U. , Nweke, P. C. , Ugbala, E. E. , Ewa, G. O. , Igwenyi, I. O. , Egwu, C. O. , Alum, E. U. , Chukwu, D. C. , & Famurewa, A. C. (2022). *Cucumeropsis mannii* seed oil (CMSO) attenuates alterations in testicular biochemistry and histology against Bisphenol A‐induced toxicity in male Wister albino rats. Heliyon, 8, e09162. 35846473 10.1016/j.heliyon.2022.e09162 PMC9280550

[fsn33260-bib-0002] Ahangarpour, A. , Zeidooni, L. , Samimi, A. , Alboghobeish, S. , Khorsandi, L. S. , & Moradi, M. (2018). Chronic exposure to arsenic and a high‐fat diet additively induced cardiotoxicity in male mice. Resource of Pharmacological Science, 13, 47–53. 10.4103/1735-5362.220967 PMC5772081 29387111

[fsn33260-bib-0003] Aja, P. M. , Nwachukwu, N. , Ibiam, A. U. , Igwenyi, I. O. , & Onu, P. N. (2014). Comparative evaluation of transaminases and alkaline phosphatase activities in albino rats administered aqueous, Ethanolic, and Methanolic extracts of *Moringa oleifera* seeds locally grown in Abakaliki, Nigeria. Journal of Biological and Chemical Research, 31(1), 164–181.

[fsn33260-bib-0004] Anjum, S. , Rahman, S. , Kaur, M. , Ahmad, F. , Rashid, H. , & Ansari, R. A. (2011). Melatonin ameliorates bisphenol A‐induced biochemical toxicity in testicular mitochondria of mice. Food and Chemical Toxicology, 49, 2849–2854. 21840368 10.1016/j.fct.2011.07.062

[fsn33260-bib-0005] Arias‐Reyes, C. , Soliz, J. , & Joseph, V. (2021). Mice and rats display different Ventilatory, hematological, and metabolic features of acclimatization to hypoxia. Frontiers in Physiology, 12, 647822. 10.3389/fphys.2021.647822 33776799 PMC7994900

[fsn33260-bib-0006] Awuchi, C. G. , & Awuchi, C. G. (2019). Physiological effects of plastic wastes on the endocrine system (Bisphenol a, phthalates, Bisphenol S, PBDEs, TBBPA). International Journal of Bioinformatics and Computational Biology, 4(2), 11–29.

[fsn33260-bib-0007] Awuchi, C. G. , & Okpala, C. O. R. (2022). Natural nutraceuticals, especially functional foods, their major bioactive components, formulation, and health benefits for disease prevention – An overview. Journal of Food Bioactives, 19, 97–123. 10.31665/JFB.2022.18317

[fsn33260-bib-0008] Awuchi, C. G. , & Twinomuhwezi, H. (2021). The medical, pharmaceutical, and nutritional biochemistry and uses of some common medicinal plants. In M. Ozturk & G. F. B. Ameenah (Eds.), Medicinal and aromatic plants of the world. Encyclopedia of Life Support Systems (EOLSS), Developed under the Auspices of UNESCO, ELOSS Publishers.

[fsn33260-bib-0070] Awuchi, C. G. (2023). Important Medicinal and Aromatic Plants – Africa. Encyclopedia of Life Support Systems (EOLSS), Developed under the Auspices of UNESCO, ELOSS Publishers.

[fsn33260-bib-0009] Badifu, G. I. O. , & Ogunsua, A. O. (1990). Chemical composition of kernels from some species of *Cucumbitaceae* grown in Nigeria. Plant Foods for Human Nutrition, 41(1), 35–44. 10.1007/BF02196380 1850132

[fsn33260-bib-0010] Basharat, Z. , Afzaal, M. , Saeed, F. , Islam, F. , Hussain, M. , Ikram, A. , Pervaiz, M. U. , & Awuchi, C. G. (2023). Nutritional and functional profile of carob bean (*Ceratonia siliqua*): A comprehensive review. International Journal of Food Properties, 26(1), 389–413. 10.1080/10942912.2022.2164590

[fsn33260-bib-0011] Bello, I. J. , Oyebode, O. T. , Olanlokun, J. O. , Omodara, T. O. , & Olorunsogo, O. O. (2021). Plumbagin induces testicular damage via mitochondrial‐dependent cell death. Chemico‐Biological Interactions, 347, 109582. 10.1016/j.cbi.2021.109582 34302802

[fsn33260-bib-0012] Biedermann, T. , Grob, F. S. , Tschudin, P. , & Grob, K. (2010). Transfer of bisphenol a from thermal printer paper to the skin. Analytical and Bioanalytical Chemistry, 398, 571–576. 20623271 10.1007/s00216-010-3936-9

[fsn33260-bib-0013] Bindhumol, V. , Chitra, K. C. , & Mathur, P. P. (2003). Bisphenol a induces reactive oxygen species generation in the liver of male rats. Toxicology, 188, 117–124. 12767684 10.1016/s0300-483x(03)00056-8

[fsn33260-bib-0014] Boubekeur, S. , Messaoudi, M. , Awuchi, C. G. , Otekunrin, O. , Sawicka, B. , Idjeri‐Mecherara, S. , Bouchareb, S. , Hassani, A. , Sharifi‐Rad, M. , Begaa, S. , & Rebiai, A. (2022). Biological properties and polyphenols content of Algerian *Cistus salviifolius* L. aerial parts. European Journal of Biological Research, 12(2), 163–180. 10.5281/zenodo.6561505

[fsn33260-bib-0015] Buege, J. A. , & Aust, S. D. (1978). Microsomal lipid peroxidation. Methods in Enzymology, 52, 302–310. 672633 10.1016/s0076-6879(78)52032-6

[fsn33260-bib-0016] Catala, A. (2009). Lipid peroxidation of membrane phospholipids generates hydroxy‐alkenals and oxidized phospholipids active in physiological and/or pathological conditions. Chemistry and Physics of Lipids, 157, 1–11. 18977338 10.1016/j.chemphyslip.2008.09.004

[fsn33260-bib-0018] Cimmino, I. , Fiory, F. , Perruolo, G. , Miele, C. , Beguinot, F. , Formisano, P. , & Oriente, F. (2020). Potential mechanisms of Bisphenol a (BPA) contributing to human disease. International Journal of Molecular Sciences, 21(16), 5761. 10.3390/ijms21165761 32796699 PMC7460848

[fsn33260-bib-0019] Dobrzyńska, M. M. , & Radzikowska, J. (2013). Genotoxicity and reproductive toxicity of bisphenol a and X‐ray bisphenol a combination in male mice. Drug and Chemical Toxicology, 36(1), 19–26. 22263531 10.3109/01480545.2011.644561

[fsn33260-bib-0020] Dror, Y. , Cohen, Y. , & Yerushalmi‐Rozen, R. (2006). Structure of gum arabic in aqueous solution. Journal of Polymer Science Part B: Polymer Physics, 44(22), 3265–3271.

[fsn33260-bib-0021] Eleazu, K. , Aja, P. M. , & Eleazu, C. O. (2021). Cocoyam (*Colocasia esculenta*) modulates some parameters of testosterone propionate induced rat model of benign prostatic hyperplasia. Drug and Chemical Toxicology, 45, 1923–1933. 10.1080/01480545.2021.1892956 33641553

[fsn33260-bib-0022] Ewere, E. G. , Okolie, N. P. , Avan, E. D. , & Umoh, P. E. (2021). Comparative effects of ethanol leaf and stem bark extracts of *Irvingia gabonensis* (BUSH MANGO) on sodium arsenite‐induced lipid profile perturbtions in wistar rats. Clinical Phytoscience, 7, 3. 10.1186/s40816-020-00241-5

[fsn33260-bib-0023] Ferrentino, G. , Morozova, K. , Horn, C. , & Scampicchio, M. (2020). Extraction of essential oils from medicinal plants and their utilization as food antioxidants. Current Pharmaceutical Design, 26(5), 519–541. 10.2174/1381612826666200121092018 31965940

[fsn33260-bib-0024] Flohe, L. , & Gunzler, W. A. (1984). Assays of glutathione peroxidase. Methods of Enzymology, 105, 114–121. 10.1016/s0076-6879(84)05015-1 6727659

[fsn33260-bib-0025] Flohe, L. , & Otting, F. (1984). Superoxide dismutase assays. Methods of Enzymology, 105, 93–104. 10.1016/s0076-6879(84)05013-8 6328209

[fsn33260-bib-0026] Garcia, R. A. , & Sajjad, H. (2022). Anatomy, abdomen and pelvis, scrotum. In StatPearls [internet]. StatPearls Publishing Retrieved 25 July, 2022, from https://www.ncbi.nlm.nih.gov/books/NBK549893/ 31751083

[fsn33260-bib-0027] Gassman, N. R. (2017). Induction of oxidative stress by bisphenol a and its pleiotropic effects. Environmental and Molecular Mutagenesis, 58(2), 60–71. 10.1002/em.22072 28181297 PMC5458620

[fsn33260-bib-0028] Geens, T. , Aerts, D. , & Berthot, C. (2012). A review of dietary and non‐dietary exposure to bisphenol‐A. Journal of Food and Chemical Toxicology, 50(10), 3725–3740. 22889897 10.1016/j.fct.2012.07.059

[fsn33260-bib-0031] Gurmeet, K. S. S. , Rosnah, I. , Normadiah, M. K. , Das, S. , & Mustafa, A. M. (2014). Detrimental effects of Bisphenol A on development and functions of the male reproductive system in experimental rats. Journal of Clinical Sciences, 13, 151–160. PMC4464354 26417249

[fsn33260-bib-0032] Gurunath, S. , Pandian, Z. , Anderson, R. A. , & Bhattacharya, S. (2011). Defining infertility, a systematic review of prevalence studies. Human Reproduction Update, 17, 575–588. 21493634 10.1093/humupd/dmr015

[fsn33260-bib-0073] Hassan, Z. K. , Elobeid, M. A. , Virk, P. , Omer, S. A. , ElAmin, M. , Daghestani, M. H. , & AlOlayan, E. M. (2012). Bisphenol A induces hepatotoxicity through oxidative stress in rat model. Oxidative Medicine and Cellular Longevity, 2012, 194829. 10.1155/2012/194829 22888396 PMC3409570

[fsn33260-bib-0033] Jain, S. , Mahendra, K. H. , Suranagi, U. D. , & Mediratta, P. K. (2011). Protective oxidative stress in rats. Food and Chemical Toxicology, 49, 1404–1409. 21440025 10.1016/j.fct.2011.03.032

[fsn33260-bib-0034] Kabuto, H. , Hasuike, S. , Minagawa, N. , & Shishibori, T. (2003). Effects of bisphenol a on the metabolisms of active oxygen species in mouse tissues. Environmental Resources, 93(1), 31–35. 10.1016/s0013-9351(03)00062-8 12865045

[fsn33260-bib-0035] Khodayar, M. J. , Kalantari, H. , Khorsandi, L. , Rashno, M. , & Zeidooni, L. (2018). Betaine protects mice against acetaminophen hepatotoxicity possibly via mitochondrial complex II and glutathione availability. Biomedicine & Pharmacotherapy, 103, 1436–1445. 29864928 10.1016/j.biopha.2018.04.154

[fsn33260-bib-0036] Koffi, K. K. , Gbotto, A. A. , Malice, M. , Dje, Y. , Bertin, P. , Bandoin, J. P. , & Zoro‐Bi, I. A. (2008). Morphology and allozyme variation in a collection of *Cucumeropsis mannii* Naudin (Cucurbitaceae) form Cote d'Ivoire. Biochemical Systematics and Ecology, 36, 777–789.

[fsn33260-bib-0037] Korkmaz, A. , Ahbab, M. A. , Kolankaya, D. , & Barlas, N. (2010). Influence of vitamin C on bisphenol Anonylphenol and octylphenol induced oxidative damages in the liver of male rats. Food and Chemical Toxicology, 48(10), 2865–2871. 20643179 10.1016/j.fct.2010.07.019

[fsn33260-bib-0039] Kortse, P. A. , & Oladiran, J. A. (2013). The effects of leaf color at fruit harvest and fruit after‐ripening duration on *Cucumeropsis mannii* seed quality. Journal of Biology, Agriculture and Healthcare, 1, 190–191.

[fsn33260-bib-0071] Leakey, R. R. B. , Tientcheu Avana, M.‐L. , Awazi, N. P. , Assogbadjo, A. E. , Mabhaudhi, T. , Hendre, P. S. , Degrande, A. , Hlahla, S. , & Manda, L. (2022). The Future of Food: Domestication and Commercialization of Indigenous Food Crops in Africa over the Third Decade (2012–2021). Sustainability, 14(4), 2355. 10.3390/su14042355

[fsn33260-bib-0040] Leslie, S. W. , Soon‐Sutton, T. L. , & Khan, M. A. B. (2022). Male infertility. In StatPearls [internet]. StatPearls Publishing Retrieved Nov 28, 2022, from https://www.ncbi.nlm.nih.gov/books/NBK562258/

[fsn33260-bib-0041] Li, D. K. , Zhou, Z. , Miao, M. , He, Y. , Wang, J. , & Ferber, J. (2012). Urine bisphenol‐A (BPA) level in relation to semen quality. Fertility Series, 95(2), 625–630. 10.1016/j.fertnstert.2010.09.026 21035116

[fsn33260-bib-0042] Marieb, E. N. , & Hoehn, K. (2014). Human anatomy and physiology (Vol. 334, 6th ed., pp. 420–530). Pearson Education Incorporated.

[fsn33260-bib-0043] Mbuli, Y. , Belitz, H. D. , Gerstenberg, H. , Kaiser, K. P. , Maniwa, K. , Medl, A. , Scherz, H. , & Weder, J. K. P. (1983). Studies on the chemical composition of the seeds from *Cucumeropsis mannii* and their suitability as a food. Journal of Lebensm Unters Forsch, 177(1), 37–40. 10.1007/BF01042494 6624267

[fsn33260-bib-0044] Meeker, J. D. , Calafat, A. M. , & Hauser, R. (2010). Urinary bisphenol concentrations in relation to serum thyroid and reproductive hormone levels in men from an infertility clinic. Environmental Science & Technology, 44(4), 1458–1463. 20030380 10.1021/es9028292 PMC2823133

[fsn33260-bib-0046] Messaoudi, M. , Rebiai, A. , Sawicka, B. , Atanassova, M. , Ouakouak, H. , Larkem, I. , Egbuna, C. , Awuchi, C. G. , Boubekeur, S. , Ferhat, M. A. , Begaa, S. , & Benchikha, N. (2022). Effect of extraction methods on polyphenols, flavonoids, mineral elements, and biological activities of essential oil and extracts of *Mentha pulegium* L. Molecules, 27(1), 11. 10.3390/molecules27010011 PMC8746320 35011242

[fsn33260-bib-0048] Mohapatra, D. , Brar, S. , Tyagi, R. , & Surampalli, R. (2010). Physicochemical pre‐treatment and biotransformation of wastewater and wastewater sludge fate of bisphenol A. Chemosphere, 78, 923–941. 20083294 10.1016/j.chemosphere.2009.12.053

[fsn33260-bib-0049] Nanjappa, M. K. , Simon, L. , & Akingbemi, B. T. (2012). The industrial chemical Bisphenol A (BPA) interferes with proliferative activity and the development of steroidogenic capacity in rat Leydig cells. Biology of Reproduction, 86(5), 135. 22302688 10.1095/biolreprod.111.095349 PMC3364919

[fsn33260-bib-0074] Nolfi‐Donegan, D. , Braganza, A. , & Shiva, S. (2020). Mitochondrial electron transport chain: Oxidative phosphorylation, oxidant production, and methods of measurement. Redox Biology, 37, 101674. 10.1016/j.redox.2020.101674 32811789 PMC7767752

[fsn33260-bib-0050] Norazit, A. , Mohamad, J. , Razak, S. A. , Abdulla, M. A. , & Azmil, A. (2012). Effects of soya bean extract bisphenol a and 17β‐estradiol on the testis and circulating levels of testosterone and estradiol among peripubertal juvenile male Sprague‐Dawley rats. SainsMalaysiana, 41(1), 63–69.

[fsn33260-bib-0051] Novo, E. , & Parola, M. (2008). Redox mechanisms in hepatic chronic wound healing and fibrogenesis. Fibrogenesis and Tissue Repair Journal, 1(1), 5. 10.1186/1755-1536-1-5 PMC2584013 19014652

[fsn33260-bib-0052] Nwozo, O. S. , Effiong, E. M. , Aja, P. M. , & Awuchi, C. G. (2023). Antioxidant, phytochemical, and therapeutic properties of medicinal plants: A review. International Journal of Food Properties, 26(1), 359–388. 10.1080/10942912.2022.2157425

[fsn33260-bib-0053] OECD . (2008). OECD: Guidelines for the testing of chemicals; acute oral toxicity: up‐and‐down procedures. OECD Publishing no. 425, Retrieved October 2008 from http://www.oecdilibrary.org/environment/test‐no‐425‐acute‐oral‐toxicity‐up‐and‐downprocedure‐9789264071049‐en

[fsn33260-bib-0054] Okwundu, O. S. , Chiama, C. J. , Chiama, C. J. , Ucheagwu, P. C. , Uzoma, E. K. , Okaro, C. A. , & Muojama, O. E. (2021). The untapped industrial crop, *Cucumeropsis mannii*: Dry oil extraction, characterization and potential use as biodiesel feedstock and heavy metal sink. Sustainable Environment Research, 31, 10. 10.1186/s42834-021-00082-y

[fsn33260-bib-0055] Olarewaju, O. O. , Fajinmi, O. O. , Arthur, G. D. , Coopoosamy, R. M. , & Naidoo, K. K. (2021). Food and medicinal relevance of Cucurbitaceae species in eastern and southern Africa. Bulletin of the National Research Centre, 45, 208. 10.1186/s42269-021-00659-y

[fsn33260-bib-0057] Park, Y. J. , & Pang, M. G. (2021). Mitochondrial functionality in male fertility: From spermatogenesis to fertilization. Antioxidants, 10(1), 98. 10.3390/antiox10010098 33445610 PMC7826524

[fsn33260-bib-0058] Ranjit, N. , Siefert, K. , & Padmanabhan, V. (2010). Bisphenol‐A and disparities in birth outcomes: A review and directions for future research. Journal of Perinatology, 30, 2–9. 19587689 10.1038/jp.2009.90 PMC4028155

[fsn33260-bib-0059] Sakaue, M. , Ohsako, S. , Ishimura, R. , Kurosawa, S. , Kurohmaru, M. , Hayashi, Y. , Aoki, Y. , Yonemoto, J. , & Tohyama, C. (2007). Bisphenol‐A affects spermatogenesis in adult rats even at a low dose. Journal of Occupational Health, 43, 185–190.

[fsn33260-bib-0060] Saleem, U. (2017). Acute oral toxicity evaluation of aqueous ethanolic extract of Saccharummunja Roxb. Roots in albino mice as per OECD 425 TG. Toxicology Reports, 4, 580–585. 29152463 10.1016/j.toxrep.2017.10.005 PMC5671618

[fsn33260-bib-0061] Santiago, J. , Silva, J. V. , Santos, M. A. S. , & Fardilha, M. (2021). Fighting Bisphenol A‐induced male infertility: The power of antioxidants. Antioxidants (Basel, Switzerland), 10(2), 289. 10.3390/antiox10020289 33671960 PMC7919053

[fsn33260-bib-0062] Sayeed, I. , Parvez, S. , Winkler‐Stuck, K. , Seitz, G. , Trieu, I. , Wallesch, C. W. , Schonfeld, P. , & Siemen, D. (2006). Patch‐clamp reveals a powerful blockade of the mitochondria permeability transition pore by the D2‐receptor agonist. Federation of American Societies for Experimental Biology Journal, 20, 556–558. 16407457 10.1096/fj.05-4748fje

[fsn33260-bib-0063] Sirasanagandla, S. R. , Al‐Huseini, I. , Sakr, H. , Moqadass, M. , Das, S. , Juliana, N. , & Abu, I. F. (2022). Natural products in mitigation of Bisphenol a toxicity: Future therapeutic use. Molecules, 27(17), 5384. 10.3390/molecules27175384 36080155 PMC9457803

[fsn33260-bib-0064] Takahashi, O. , & Oishi, S. (2003). Testicular toxicity of dietarily or parenterally administered bisphenol A in rats and mice. Food and Chemical Toxicology, 41, 1035–1044. 12804662 10.1016/s0278-6915(03)00031-0

[fsn33260-bib-0065] Trusca, V. G. , Dumitrescu, M. , Fenyo, I. M. , Tudorache, I. F. , Simionescu, M. , & Gafencu, A. V. (2019). The mechanism of Bisphenol A Atherogenicity involves Apolipoprotein A‐I downregulation through NF‐κB activation. International Journal of Molecular Sciences, 20(24), 6281. 10.3390/ijms20246281 31842455 PMC6941038

[fsn33260-bib-0066] Turrens, J. F. (2003). Mitochondrial formation of reactive oxygen species. The Journal of Physiology, 552(2), 335–444. 14561818 10.1113/jphysiol.2003.049478 PMC2343396

[fsn33260-bib-0072] Umapathy, A. , Li, B. , Donaldson, P. J. , & Lim, J. C. (2018). Functional characterisation of glutathione export from the rat lens. Experimental Eye Research, 166, 151–159. 10.1016/j.exer.2017.10.010 29032155

[fsn33260-bib-0067] WHO . (2021). WHO laboratory manual for the examination and processing of human semen. World Health Organization Retrieved January 13, 2023 from https://www.who.int/publications/i/item/9789240030787

[fsn33260-bib-0068] Wu, J. H. , Jiang, X. R. , Liu, G. M. , Liu, X. Y. , He, G. L. , & Sun, Z. Y. (2011). Oral exposure to low dose bisphenol a aggravates testosterone‐induced benign hyperplasia prostate in rats. Toxicology and Industrial Health, 27(9), 810–819. 21415097 10.1177/0748233711399310

[fsn33260-bib-0069] Zahnit, W. , Smara, O. , Bechki, L. , Souici, C. B. , Messaoudi, M. , Benchikha, N. , Larkem, I. , Awuchi, C. G. , Sawicka, B. , & Simal‐Gandara, J. (2022). Phytochemical profiling, mineral elements, and biological activities of *Artemisia campestris* L. Grown in Algeria. Horticulturae, 8(10), 914. 10.3390/horticulturae8100914

